# The Neural Mechanisms of Group Membership Effect on Emotional Mimicry: A Multimodal Study Combining Electromyography and Electroencephalography

**DOI:** 10.3390/brainsci14010025

**Published:** 2023-12-25

**Authors:** Beibei Kuang, Shenli Peng, Yuhang Wu, Ying Chen, Ping Hu

**Affiliations:** 1 College of International Relations, National University of Defense Technology, Nanjing 210039, China; 2 Department of Psychology, Renmin University of China, Beijing 100872, China; wuyuhang@ruc.edu.cn (Y.W.); chenying2019@ruc.edu.cn (Y.C.); 3 College of Education, Hunan Agricultural University, Changsha 410128, China; slpeng@hunau.edu.cn

**Keywords:** emotional mimicry, group membership, in-group preference, neural mechanism, ERP difference

## Abstract

Emotional mimicry plays a vital role in understanding others’ emotions and has been found to be modulated by social contexts, especially group membership. However, the neural mechanisms underlying this modulation remain unclear. We explored whether and how group membership modulated emotional mimicry using a multimodal method combining facial electromyography (fEMG) and electroencephalography (EEG). We instructed participants to passively view dynamic emotional faces (happy vs. angry) of others (in-group vs. out-group) and simultaneously recorded their fEMG and EEG responses. Then, we conducted combined analyses of fEMG-EEG by splitting the EEG trials into two mimicry intensity categories (high-intensity mimicry vs. low-intensity mimicry) according to fEMG activity. The fEMG results confirmed the occurrence of emotional mimicry in the present study but failed to find a group membership effect. However, the EEG results showed that participants mimicked in-group happiness and anger more than out-group. Importantly, this in-group preference involved different neural mechanisms in happiness and anger mimicry. In-group preference for happiness mimicry occurred at multiple neural mechanisms such as N1 (at P7, Pz, and P8), P2 (at Pz and P8), N2 (at P8), and P3 (at P7, Pz, and P8); in-group preference for anger mimicry occurred at P1 (at P7) and P2 (at Pz). Our findings provide new neural evidence for the effect of group membership on emotional mimicry by uncovering the temporal dynamics of this effect.

## 1. Introduction

Emotional mimicry refers to the rapid imitation of others’ emotional expressions during social interactions and plays an important role in understanding others’ emotions [1,2]. It occurs throughout the lifespan [3,4,5,6,7]. The present study focused on facial emotional mimicry, which arouses facial muscle activity and is often measured by the facial electromyography (fEMG) technique [8]. Studies have found that emotional mimicry is modulated by social context [9,10,11,12], of which the expresser’s group membership (in-group vs. out-group) is an important factor. Many previous studies have investigated how group membership modulates emotional mimicry [13,14]; however, they have reported inconsistent results and rarely focused on the neural mechanisms underlying this modulation. The present study focused on the neural mechanisms by which group membership impacted emotional mimicry by a multimodal method of fEMG and electroencephalography (EEG).

Social groups are basic units of social life. Belonging to a certain group and being accepted by the group members are fundamental needs for individuals [15]. To fulfill the need for belonging, individuals always take opportunities to join various groups and become group members. Therefore, group membership is an important social cue for social interactions between individuals. The present study focused on group membership information derived from facial expressions. According to social identity theory [16] and self-category theory [17], group members share a social identity. Their “individuality” is de-emphasized, and their perceptions of self-concept are more likely based on the whole group membership. To maintain a positive self-concept, they always show in-group preference and out-group bias [18,19]. For example, researchers have discovered that individuals exhibit more positive attributions, emotions, and behaviors toward in-group members than toward out-group members [20,21,22]. Furthermore, EEG and functional magnetic resonance imaging (fMRI) studies have found that individuals show different neural activity in response to in-group members and out-group strangers [21,23,24,25]. Thus, as an important social context, group membership affects both the behavioral and neural responses of individuals.

Many studies have explored the effect of group membership on emotional mimicry using the fEMG technique, Facial Action Coding System, or other behavioral methods [13,14,26,27]; however, their results have been inconsistent and even contradictory. For example, Bourgeois and Hess [13] found that participants mimicked in-group negative emotions (anger and sadness) but not the out-group negative emotions, while they mimicked happy displays regardless of the expresser’s group membership. Van der Schalk et al. [14] found that participants mimicked in-group anger and fear more than out-group, while neither mimicked in-group nor out-group happiness. Sachisthal et al. [27] found that participants mimicked both in-group and out-group happiness; however, for negative emotions (anger, fear, and sadness), they failed to find any emotional mimicry or group membership effects. In summary, neither study identified a group membership effect on happiness mimicry. Regarding negative emotional mimicry, Bourgeois and Hess [13] and Van der Schalk et al. [14] found an in-group preference, although Sachisthal et al. [27] failed to find any group membership effect. Additionally, other researchers found an out-group preference in anger mimicry [28]. That is, participants performed enhanced mimicry to angry out-group faces than to angry in-group faces.

The possible reasons for inconsistent results among previous studies are related to differences in research stimuli and tasks, as well as measurement and calculation methods of emotional mimicry. More importantly, these studies mainly measured facial muscular activity with fEMG and neglected to measure neural activity, so the neural mechanism of group membership effect on emotional mimicry is unknown. Furthermore, fEMG studies mainly use summed facial muscular activity in the whole stimuli duration as an index of emotional mimicry, while EEG studies often process data on all time points and can obtain time-course information of the neural activity of emotional mimicry. Therefore, the latter might be more delicate than the former in uncovering group membership effects on emotional mimicry. According to previous studies [29,30], a multimodal method of fEMG and EEG can help us investigate the neural process by which social contexts modulate emotional mimicry. Thus, the present study aimed to explore whether and how group membership modulates emotional mimicry by uncovering the facial muscular and neural variation processes involved in this modulation through the combined use of fEMG and EEG.

To our best knowledge, only one study investigated neural mechanisms by which group membership modulated emotional mimicry with fMRI [31]. This study is enlightening, but the temporal dynamics of this modulation remain unclear because fMRI provides a low time resolution. They proposed that a more time-sensitive measure, such as the event-related brain potential (ERP), might tap into the uncertainty. To investigate the temporal dynamics of emotional mimicry, Achaibou et al. [29] recorded fEMG and scalp EEG simultaneously when participants observed happy or angry expressions, providing the basis for our exploration. According to the fEMG activity recorded for each muscle in any given trial, they conducted multimodal analysis by dividing all EEG trials into two types: high-intensity mimicry (HIM) and low-intensity mimicry (LIM). By contrasting the EEG activity of HIM and LIM, they discovered ERPs involved in emotional mimicry. Unfortunately, Achaibou et al. [29] did not include social contextual information. Based on this method, Kuang et al. [30] further explored how the eye-gaze direction regulated emotional mimicry. Since the information on eye-gaze direction cannot represent all kinds of social contexts, the neural process for the group membership effect on emotional mimicry might be completely different. Thus, using this multimodal method of integrating fEMG and EEG, the present study investigated the temporal dynamics of facial muscular and neural activity of group membership effects on emotional mimicry.

In short, emotional mimicry plays a vital role in social life and is influenced by social context. As an important social context, group membership has traditionally been believed to elicit in-group preference and out-group hate. However, researchers have not reached a consensus on whether group membership modulates emotional mimicry and have neglected to investigate the neural mechanisms of this modulation. This study aimed to explore whether and how group membership impacts emotional mimicry. Since happiness and anger represent typical positive and negative expressions, respectively, the present study focused on happiness and anger mimicry and used happy and angry faces as stimulus materials. To fulfill this purpose, we recorded fEMG and EEG activity in participants while observing the emotional faces (happiness vs. anger) of others (in-group vs. out-group). Thus, the present study used a within-subject design of emotion (happy vs. angry) × group (in-group vs. out-group). Considering the inconsistent results in previous studies, we formulated open hypotheses in the present study. For the fEMG data, we first confirmed whether emotional mimicry occurred in the present study, and then explored how group membership modulated emotional mimicry by conducting a time-course analysis. For the EEG data, we explored the neural mechanisms through which group membership regulates emotional mimicry by conducting a combined analysis of fEMG and EEG. This combined analysis included three steps: first, EEG trials were divided into two types (HIM vs. LIM) based on fEMG activity; second, neural signals specific to emotional mimicry were extracted by subtracting the ERP of the LIM from that of the HIM; finally, by applying the HIM vs. LIM ERP difference as a dependent variable, we explored the neural mechanisms underlying the effect of group membership on emotional mimicry.

## 2. Materials and Methods

### 2.1. Participants

We recruited participants through the campus Bulletin Board System. Participants without a history of mental disorder, with normal or corrected-to-normal vision were included. Thirty healthy Chinese college students volunteered to participate in the experiment. All participants reported normal or corrected-to-normal vision and provided written informed consent prior to the experiment. They were informed that they could withdraw from the study at any time and were paid for their participation. Two of them were excluded for excessive noise after a visual inspection. According to previous studies, participants who exhibited artifacts in more than 25% of trials were excluded [32]. In our research, two participants exhibited artifacts on more than 25% of trials because of bridges between electrodes due to conductive gel. Thus, the two participants were excluded and a total of 28 participants (18 women and 10 men; mean age: 20.17 ± 2.01 years) were included in the analyses. The ethics board of the authors’ institution approved this project.

### 2.2. Materials

**Figure 1:**
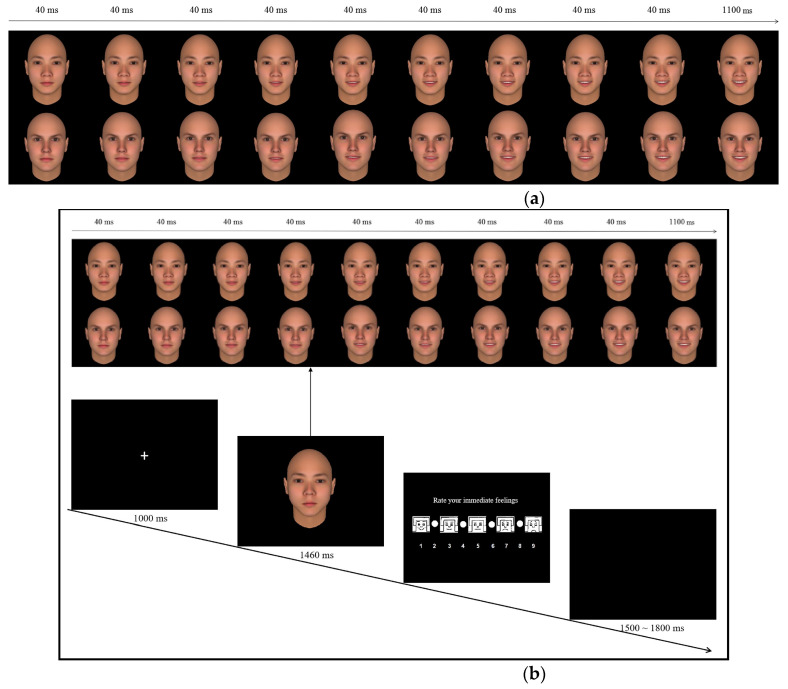
(**a**) Dynamic expression of happy faces. Examples of the time frames used for happy face stimuli of the in-group (upper part) and out-group (lower part). (**b**) Procedure of the present experiment.

In total, 20 neutral Asian Chinese faces (in-group; 10 women, 10 men) and 20 neutral Western faces (out-group; 10 women, 10 men) were selected from the Chinese Affective Picture System [33] and NimStim [34], respectively. Each face identity was morphed between a neutral expression and a typical expression (happy or angry) using FACSGen software 2.0 [35], leading to a sequence of 10 frames per face with increasing emotional intensity (0%, 15%, 30%, 45%, 60%, 70%, 80%, 90%, 100%, and 110% intensity). A total of 40 face sequences were created for each emotion (happy or angry), consisting of half in-group and half out-group sequences. These face sequences were presented sequentially from 0% to 110% intensity using E-prime. In each sequence, the first nine frames continued for 40 ms each, and the last frame continued for 1100 ms (Figure 1a), producing a 1460 ms emotional dynamic clip. Thus, a total of 80 emotional clips were created. Each clip was repeated five times. Finally, 400 trials were obtained: 100 in-group happy, 100 out-group happy, 100 in-group angry, and 100 out-group angry trials.

### 2.3. Procedure

The experiment was conducted in a quiet, bright room. Participants were told that this study aimed to test their feelings toward some pictures (our research materials), so they were blind to our research purpose. After obtaining informed consent, participants were instrumented with a non-invasive EEG cap and four fEMG electrodes, and then passively viewed 400 experimental trials randomly presented on a computer screen. They were then given a rest interval after every 50 trials. Each trial consisted of a central cross-fixation (1000 ms), an emotional clip (1460 ms) at the screen center, and the self-assessment manikin scale (SAM) [36] (Figure 1b). On this scale, participants were required to rate their immediate feelings after seeing each emotional clip (9-point scale: 1 = the happiest, 5 = neutral, 9 = the angriest). The scale was set to ensure task engagement and was not analyzed. During the entire trial, the participants were instructed to focus on the central point and avoid eye blinks, saccades, and head movements. The inter-trial interval (ITI) varied from 1500 to 1800 ms. The whole experiment lasted about 90 min.

### 2.4. Data Acquisition, Processing, and Analyses

All data were acquired using the BIOSEMI Active-Two amplifier system, processed in MATLAB 2013b, and analyzed using SPSS 22.0.

#### 2.4.1. fEMG

The activity of fEMG was recorded on the left side of the participants’ faces by four active electrodes of BIOSEMI Active-Two amplifier system at 2048 Hz, with a band-pass filter of 0.1–417 Hz (https://www.biosemi.com/index.htm (accessed on 19 December 2023)), and the Common Mode Sense active electrode and the Driven Right Leg passive electrode were used as the reference and ground electrodes, respectively (https://www.biosemi.com/faq/cms&drl.htm (accessed on 19 December 2023)). Two active electrodes were placed on the zygomaticus (ZM) muscle region, and the other two were placed on the corrugator (CS) muscle region according to guidelines [37,38]. ZM and CS activity were obtained from the electrode pairs placed on each muscle by subtracting the activity of one electrode from that of the other electrode nearby. The notch filter was automatically applied by the BIOSEMI Active-Two amplifier system.

Raw fEMG data were preprocessed prior to data analysis. The digital filter range went from 20 to 400 Hz. The time window for the fEMG analysis of each trial ranged from 1000 ms pre- to 1400 ms post-stimulus. To determine the magnitude of the fEMG signal (from 0–1400 ms), the root mean square (rms) was calculated for each trial using a 100 ms interval bin. After baseline correction (from −1000 ms to stimulus onset), the fEMG activity was *z*-transformed within the subject [13,39]. Trials in which the difference in activity of two adjacent time-bins was superior to 3.5 standard deviations of the mean value for the whole trial were rejected [29]. On average, 6% of the trials were rejected in the happy condition and 7% in the angry condition.

We first performed a repeated-measures ANOVA (rmANOVA) of muscle (ZM vs. CS) × emotion (happy vs. angry) on the aggregated fEMG activity. A significant interaction effect indicated the occurrence of emotional mimicry [13,40]. Specifically, significantly higher levels of the ZM than the CS characterize emotional mimicry to happiness (EMH), while a reversed pattern is expected for emotional mimicry to anger (EMA). Considering the different fEMG activity patterns of EMH and EMA, we explored the group membership effect on EMH and EMA separately by performing an rmANOVA of muscle (ZM vs. CS) × group (in-group vs. out-group) × time-bin (14 levels: 1, …, 14). Bonferroni’s correction was applied in all multiple comparisons.

#### 2.4.2. EEG

EEG data were collected using an EEG cap with 32 active electrodes from the BIOSEMI Active-Two amplifier system with the same reference and ground electrodes, filter parameters, and acquisition rates as fEMG. The participants were equipped with these electrodes using an extended 10–20 EEG system [41].

Before data analysis, raw EEG data were preprocessed. They were resampled to 500 Hz, bandpass-filtered from 1 to 40 Hz, and segmented from 100 ms pre- to 360 ms post-stimulus. Baseline correction was performed using baseline subtraction (from −100–0 ms). Visual inspection was performed to detect noisy trials. Eye blinks and muscle artifacts were corrected using independent component analysis. Trials with an amplitude exceeding ±80 μV were automatically removed. Trials rejected by the fEMG analysis were automatically excluded from the EEG analysis.

According to visual inspection of ERP grand averages, five ERPs of interest were examined: P1, N1, P2, N2, and P3. For each of these, we selected specific electrodes and time ranges on peak amplitude observed on the mean ERPs computed in all trials. We measured all ERPs at P7, Pz, and P8. The latency and time range of P1, N1, P2, N2, and P3 were 119 ms (110–128 ms), 185 ms (176–194 ms), 243 ms (234–252 ms), 301 ms (292–310 ms), and 351 ms (342–360 ms). Within the temporal range around the peak amplitude, we calculated the mean activity for each ERP at each electrode.

Previous studies have shown that muscle and brain responses are synchronous during emotional mimicry [30,42,43]. To extract ERPs specific to emotional mimicry, EEG trials in each experimental condition were divided into two groups (HIM vs. LIM) within participant based on fEMG activity recorded for each muscle in any given trial [29]. Trials in the happy condition were divided based on ZM activity (above or below the median of the corresponding conditions). Trials in the angry condition were divided based on CS activity. For each condition, trials above the median split were included in the HIM group, whereas trials below the median split were included in the LIM group. Finally, we obtained eight new conditions: 2 (group: in-group vs. out-group) × 2 (mimicry intensity: HIM vs. LIM) × 2 (emotion: happy vs. angry). The trial numbers for each condition are listed in Table 1. All trials were averaged for each condition to obtain ERPs. ERPs specific to emotional mimicry were then isolated by subtracting the ERP of LIM from those of HIM: HIM vs. LIM ERP difference. This within-participant divide can exclude other individual factors from difference waves [44], providing a relatively pure measure of the neural correlates of emotional mimicry. Then, the neural processes of emotional mimicry in different experimental conditions can be compared.

To explore the neural mechanisms of how group membership regulates emotional mimicry, by applying the HIM vs. LIM ERP difference wave on P1, P2, N2, and P3 as the outcome measure of emotional mimicry, we conducted rmANOVAs of group (in-group vs. out-group) × emotion (happy vs. angry) at the three selected electrodes. Because this study was not concerned with the effect of location, the EEG data at the three selected electrodes were analyzed separately. Bonferroni’s correction was applied in all multiple comparisons.

## 3. Results

### 3.1. fEMG Results

Before investigating the effect of group membership on emotional mimicry, it was necessary to verify that emotional mimicry did occur in this study. Thus, we conducted an rmANOVA of emotion × muscle on fEMG data. A significant interaction effect was found (*F* (1,27) = 36.09, *p* < 0.001, *η*^2^*_p_*= 0.57), confirming the occurrence of emotional mimicry. Further simple effect analysis showed that ZM activity was stronger than CS activity in the happy condition (*M_zm-cs_* = 0.54, *p* < 0.001), whereas CS activity was stronger than ZM activity (*M_cs-zm_* = 0.59, *p* < 0.001) in the angry condition. Thus, both EMH and EMA occurred. Since EMH and EMA induced different facial muscular activity patterns, we next explored the effect of group membership on EMH and EMA separately.

**Figure 2:**
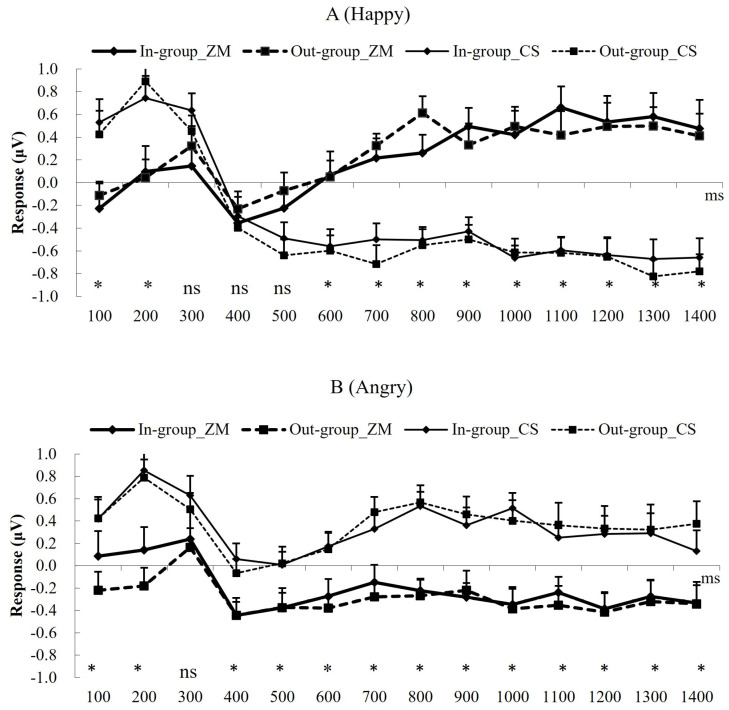
EMG responses after the stimulus onset. (**A**) Time course for EMG response from 100 to 1400 ms for happy faces. (**B**) Time course for EMG response from 100 to 1400 ms for angry faces. * denotes a significant difference between ZM and CS responses, and ns indicates a nonsignificant difference between in-group and out-group conditions. Error bars indicate standard errors.

To explore whether group membership affects emotional mimicry or not, we conducted separate rmANOVA of muscle × group × time-bin on the fEMG data in the happy and angry conditions. Results detected significant interaction effects of muscle × time-bin in both happy and angry conditions (*F* (13,15) = 4.41, *p* = 0.004, *η*^2^*_p_*= 0.79; *F* (13,15) = 2.66, *p* = 0.036, *η*^2^*_p_*= 0.70), indicating that facial muscle activity changed with time. The time course of emotional mimicry refers to Figure 2 and Table 2. However, neither main effects nor interaction effects involving group were found (in happy condition: all *F* < 1.00, *p* > 0.500; in angry condition: all *F* < 2.50, *p* > 0.100). Additional details are shown in Figure 2 and Table 3.

Herein, the fEMG data failed to identify a group membership effect. Therefore, a combined “fEMG-EEG” indicator was employed to further explore this issue. This indicator provides dependable information on emotional mimicry at the physiological level, which is linked to the neural level.

### 3.2. Neural Mechanisms of the Effect of Group Membership on Emotional Mimicry

**Figure 3:**
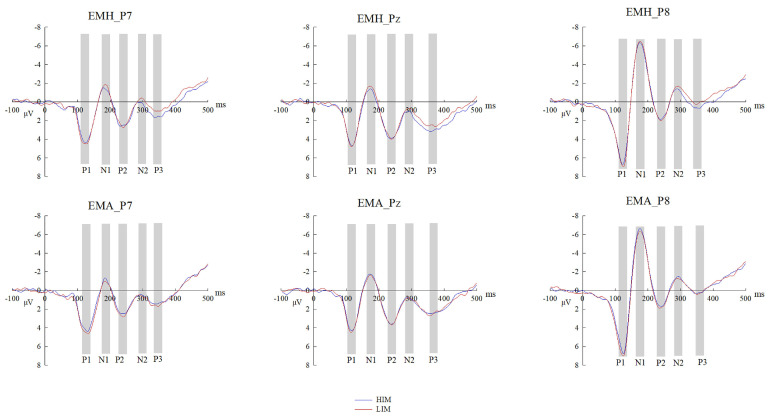
Grand average ERPs. Grand average ERPs for P1, N1, P2, N2, and P3 in HIM vs. LIM trials in each condition at three selected electrodes. EMH = emotional mimicry to happy faces. EMA = emotional mimicry to angry faces. HIM = high intensity mimicry, LIM = low intensity mimicry.

**Figure 4:**
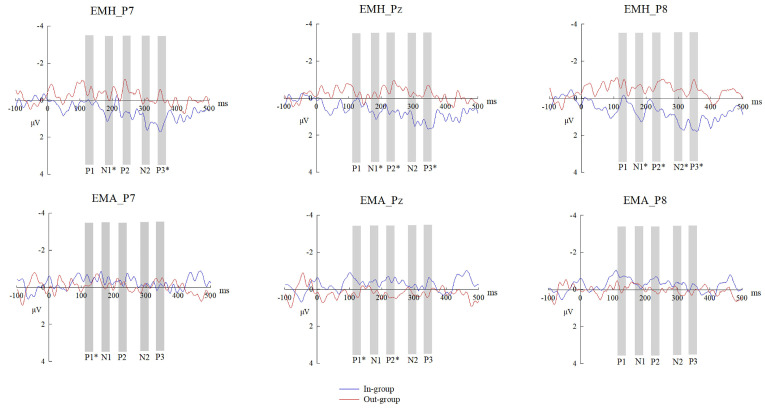
HIM vs. LIM ERP difference waves. Grand average HIM vs. LIM ERP difference waves in each condition at three selected electrodes, showing brain response differences to emotional faces when fEMG revealed high vs. low muscle activity in response to emotional expressions. * indicates a significant ERP difference between the in-group and out-group conditions. EMH = emotional mimicry to happy faces. EMA = emotional mimicry to angry faces.

**Figure 5:**
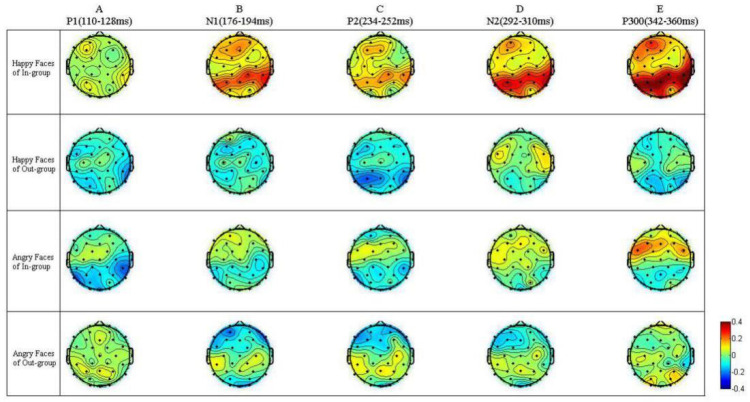
Topographical maps of HIM vs. LIM ERP difference in each condition. Scalp distribution of the electrical activity of HIM vs. LIM ERP difference under the four experimental conditions. HIM = high intensity mimicry, LIM = low intensity mimicry.

With HIM vs. LIM ERP differences on P1, N1, P2, N2, and P3 as neural indexes of emotional mimicry, we continued to explore the neural mechanisms of the group membership effect on emotional mimicry by conducting the rmANOVA of emotion × group at the three selected electrodes separately. Figure 3, Figure 4 and Figure 5 show the HIM vs. LIM ERP differences and their topographical maps for each condition.

**Figure 6:**
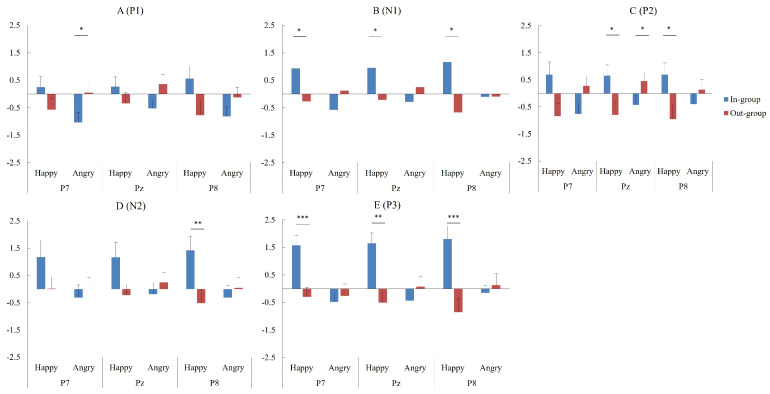
Effects of group membership on emotional mimicry for five ERP components. * *p* < 0.05, ** *p* < 0.01, *** *p* < 0.001, Error bars indicate standard errors.

On P1, the interaction effect of emotion × group was significant at P7 and P8 (*F* (1,27) = 4.83, *p* = 0.037, *η*^2^*_p_*= 0.15; *F* (1,27) = 5.21, *p* = 0.031, *η*^2^*_p_*= 0.16). At Pz, neither an interaction effect nor a main effect was found for the group (all *F* < 3.50, *p* > 0.080). Specifically, at P7, simple effect analysis revealed that out-group faces elicited larger P1 difference wave than in-group faces in the angry condition (*M_D_* = 1.08, *p* = 0.025) but not in the happy condition (*M_D_* = −0.83, *p* = 0.218). At P8, simple effect analysis failed to find a significant group membership effect in neither happy nor angry condition (all *M_D_* < 1.50, *p* > 0.080). Thus, the results of P1 detected a group membership effect on the neural process of EMA at P7, not on EMH (Figure 6A). Therefore, group membership may impact the neural process of emotional mimicry from 100 ms post-stimulus.

On N1, the interaction effect of emotion × group was significant at P7, Pz, and P8 (*F* (1,27) = 5.89, *p* = 0.022, *η*^2^*_p_*= 0.18; *F* (1,27) = 4.53, *p* = 0.043, *η*^2^*_p_*= 0.14; *F* (1,27) = 6.96, *p* = 0.014, *η*^2^*_p_*= 0.21). Simple effect analysis revealed that in-group faces evoked larger (less negative) N1 difference waves than out-group faces in the happy condition at P7, Pz, and P8 (*M_D_* = 1.20, *p* = 0.020; *M_D_* = 1.16, *p* = 0.040; *M_D_* = 1.84, *p* = 0.001), but not in the angry condition (*M_D_* = −0.70, *p* = 0.212; *M_D_* = −0.54, *p* = 0.317; *M_D_* = −0.02, *p* = 0.974). Thus, the results of N1 detected a group membership effect on the neural process of EMH at P7, Pz, and P8, not on EMA (Figure 6B).

On P2, we detected a significant interaction effect of emotion × group at the three selected electrodes (P7: *F* (1,27) = 5.81, *p* = 0.023, *η*^2^*_p_*= 0.18; Pz: *F* (1,27) = 8.80, *p* = 0.006, *η^2^_p_* = 0.25; P8: *F* (1,27) = 8.09, *p* = 0.008, *η*^2^*_p_*= 0.23). Simple effect analysis revealed that the P2 difference waves of in-group faces were larger than those of out-group faces in the happy condition at Pz and P8 (*M_D_* = 1.45, *p* = 0.036; *M_D_* = 1.64, *p* = 0.043), but not at P7 (*M_D_* = 1.53, *p* = 0.054). In the angry state, the P2 difference wave of the out-group faces was larger than that of the in-group faces at Pz (*M_D_* = 0.88, *p* = 0.049), but not at P7 and P8 (*M_D_* = 1.04, *p* = 0.079; *M_D_* = 0.53, *p* = 0.210). Thus, the results of P2 detected a group membership effect on the neural process of EMH at P7, Pz, and P8, on EMA at Pz (Figure 6C).

On N2, the interaction effect of emotion × group was significant at P8, *F* (1,27) = 7.29, *p* = 0.012, *η*^2^*_p_*= 0.21. Simple effect analysis revealed that in-group faces evoked larger (less negative) N2 difference wave than out-group faces in the happy condition at P8 (*M_D_* = 1.93, *p* = 0.008), but not in the angry condition (*M_D_* = −0.36, *p* = 0.537). At P7 and Pz, neither interaction effects nor main effects involving group were found (P7: all *F* < 2.00, *p* > 0.200; Pz: all *F* < 3.50, *p* > 0.080). Therefore, the results of N2 only showed a group membership effect on the neural process of EMH at P8, not on EMA (Figure 6D).

On P3, we found a significant interaction effect of emotion × group at the three selected electrodes (P7: *F* (1,27) = 10.34, *p* = 0.003, *η*^2^*_p_*= 0.28; Pz: *F* (1,27) = 10.03, *p* = 0.004, *η*^2^*_p_*= 0.27; P8: *F* (1,27) = 12.84, *p* = 0.001, *η*^2^*_p_*= 0.32). Simple effect analysis found that the P3 difference waves of in-group faces were larger than those of out-group faces in the happy condition at P7, Pz, and P8 (*M_D_* = 1.87, *p* = 0.001; *M_D_* = 2.16, *p* = 0.002; *M_D_* = 2.66, *p* = 0.001), but not in the angry condition (*M_D_* = −0.22, *p* = 0.693; *M_D_* = −0.51, *p* = 0.303; *M_D_* = −0.28, *p* = 0.586). Like the results of N2, the results of P3 showed a group membership effect on the neural process of EMH at P7, Pz, and P8, not on EMA (Figure 6E).

In summary, group membership modulated the neural process of emotional mimicry, and this modulation was different in happy and angry conditions. That is, the neural mechanisms by which group membership influences EMH and EMA are different. For EMH, in-group faces elicited larger N1, P2, N2, and P3 difference waves than out-group faces. For EMA, out-group faces elicited larger P1 and P2 difference waves than in-group faces. In the happy condition, a stronger neural response was related to HIM; in the angry condition, a stronger neural response was related to LIM (Figure 3). Thus, participants performed in-group preferences in both happy and angry conditions.

## 4. Discussion

The present study aimed to examine whether and how group membership impacts emotional mimicry by identifying facial muscular and neural processes during emotional mimicry using a multimodal method combining fEMG and EEG. The fEMG data replicated previous studies by showing the occurrence of happiness and anger mimicry but failed to detect any group membership effect on emotional mimicry. However, the EEG data confirmed that group membership affected both EMH and EMA. Specifically, participants performed in-group preferences during both EMH and EMA. Furthermore, the group membership effects on EMH and EMA were related to different neural mechanisms. Specifically, the effect of group membership on EMH mainly involved N1 (at P7, Pz, and P8), P2 (at Pz and P8), N2 (at P8), and P3 (at P7, Pz, and P8), whereas the effect of group membership on EMA involved P1 (at P7) and P2 (at Pz). The present study provides new neural evidence for the impact of group membership on emotional mimicry.

The fEMG data in the present study failed to identify a group membership effect on either EMH or EMA. The result of EMH was consistent with previous studies, e.g., Bourgeois and Hess [13]. The result of the EMA was consistent with Sachisthal et al.’s study [27], but it was inconsistent with Bourgeois and Hess’s and Van der Schalk et al.’s studies [13,14]. However, the EEG data in the present study showed that participants mimicked in-group happiness and anger more than out-group (in-group preferences), which broadens the current fEMG literature by providing new neural evidence in this area. These findings suggest that the fEMG data could not reflect this group membership effect, while EEG data might be more sensitive. A possible reason for this is that fEMG and EEG data have different analytical paradigms, although they are both physiological electrical signals. EEG data can be analyzed at all time points, so EEG studies can obtain continuous data. As for fEMG data, to determine the magnitude of the fEMG signal and conduct comparisons among different experimental conditions, rms analysis is always conducted using a 100 ms interval bin in the current literature. Therefore, fEMG studies only obtain point data. Continuous data catch more information than point data. Therefore, it is not strange that only EEG data detected a group membership effect in the present study. More sensitive fEMG data analysis methods should be explored in the future. Another possible reason is that these two signals have different attributes.

In-group preference has already been found in many other areas, such as emotional recognition and visual attention [45,46]. According to social identity theory [16,17], in-group preference is derived from self-categorization and depersonalization. The former explains how individuals interpret the social world as a combination of in- and out-groups. The latter explains how individuals become integrated into a group when they categorize themselves as in-group members. After that, individuals show in-group preference to create or maintain a positive social identity and self-esteem [47]. Moreover, emotional mimicry serves an affiliative function in social interactions [48,49,50]. Enhanced emotional mimicry may be an effective way to achieve positive self-esteem.

Our EEG data also revealed that group membership affected five stages of face processing: P1, N1, P2, N2, and P3, which is consistent with previous studies on emotional face perception and recognition areas [51,52,53]. Specifically for face processing, P1 is generally related to automatic perception and sensitive to the physical characteristics of faces [54]; N1 is believed to be the index of a discrimination process [55]; P2 often reflects featural face processing [56]; N2 is believed to reflect the emotional value of faces [57]; and P3 is believed to be a conscious and cognitive component related to the social cognition process [58]. Therefore, emotional mimicry might be built into the face-processing process.

It should be noted that, in the present study, the group membership effect on EMH mainly occurred at N1, P2, N2, and P3, while the group membership effect on EMA only occurred at P1 and P2. The effect of group membership on EMA is earlier than that on EMH. This phenomenon can be understood in terms of the negative bias found in the evolutionary and developmental literature [59]. Negative information, for instance, angry faces, means threat or risk. Positive information, for instance, happy faces, is related to the resource development function. Negative bias helps people survive by recognizing risks quickly. Thus, individuals are more sensitive to angry faces and respond to angry faces earlier. However, they downregulate or suppress their neural responses to anger in the later conscious stage to relieve discomfort from threatening information. Therefore, the group membership effect on EMA disappears after P2.

## 5. Conclusions

In summary, our study employed a multimodal analysis by combining fEMG and EEG to uncover whether, when, and how group membership modulates emotional mimicry. Our results verified that individuals performed in-group preferences when both happy and angry faces were mimicked, and these in-group preferences were reflected in five face processing stages: P1, N1, P2, N2, and P3. Importantly, in-group preference for anger mimicry occurred earlier than that for happiness mimicry, indicating different neural pathways of group membership effects on happiness and anger mimicry. This is the first study to explore the temporal dynamics and neural process of group membership effect on emotional mimicry; it is supplemental to previous inconsistent fEMG studies. These findings provide new neural evidence for the effect of group membership on emotional mimicry and broaden our understanding of social cognition processes. That is, the emotion transmission process in social interaction might be modulated by group membership or emotion valence.

Since emotional mimicry is hard to manipulate as an independent variable, its neural process is difficult to measure directly. This study employed the HMI vs. LMI ERP difference wave method to extract neural activity specific to emotional mimicry, providing a more delicate instrument to measure emotional mimicry, and providing chances to explore the neural mechanisms by which group membership modulates emotional mimicry. In the future, the application of multimodal measurements will be useful and necessary to uncover the neural mechanisms underlying many more interesting phenomena in this area.

## 6. Limitations

However, there are several limitations in the present study. First, these findings may be limited to the current stimulus type; therefore, more investigations in other social contexts are needed. Second, we did not assess how much participants (Chinese college students in the present study) identified Chinese faces as in-group members and how distanced they considered themselves from Western people; future studies should address such information and take them as controlled variables. Third, these conclusions should be interpreted with caution because the present study was conducted among Chinese college students. The process of emotional mimicry might be different in other cultural contexts or other age groups. Future studies can consider culture or age group difference in this research area.

## Figures and Tables

**Table 1:** Trial distribution in four conditions (emotion × group × mimicry intensity).

	Trials of Happy Faces		Trials of Angry Faces	
	In-Group	Out-Group	In-Group	Out-Group
HIM	48 trials	47 trials	46 trials	47 trials
LIM	48 trials	47 trials	46 trials	47 trials

HIM = high intensity mimicry, LIM = low intensity mimicry.

**Table 2:** Pairwise contrasts in the difference between ZM and CS activity at 14 time-bins.

Emotional Mimicry	Pairwise Contrasts	T1	T2	T3	T4	T5	T6	T7	T8	T9	T10	T11	T12	T13	T14
EMH	*M*_ZM-CS_	−0.70	−0.80	−0.25	−0.10	0.30	0.59	0.83	0.99	0.81	1.03	1.21	1.26	1.41	1.30
	*p*	0.002	0.002	0.255	0.621	0.098	0.004	<0.001	<0.001	0.001	<0.001	<0.001	<0.001	<0.001	<0.001
EMA	*M*_ZM-CS_	−0.56	−0.81	−0.21	−0.57	−0.45	−0.54	−0.62	−0.82	−0.64	−0.85	−0.63	−0.73	−0.55	−0.57
	*p*	0.009	<0.001	0.337	<0.001	0.004	0.001	0.001	<0.001	0.004	0.001	0.005	0.001	0.024	0.012

Note. T1–T14: 100 ms time-bins from 100 to 1400 ms post-stimulus. EMH = emotional mimicry to happy faces. EMA = emotional mimicry to angry faces. *M_ZM-CS_* = the mean difference between ZM and CS activity.

**Table 3:** Pairwise contrasts in the difference between in-group and out-group conditions at 14 time-bins.

Emotional Mimicry	Pairwise Contrasts	T1	T2	T3	T4	T5	T6	T7	T8	T9	T10	T11	T12	T13	T14
EMH	*M*_IN-OUT_	−0.09	0.15	−0.15	−0.05	−0.16	0.10	−0.13	−0.41	0.18	0.01	0.29	0.04	0.11	0.07
	*p*	0.665	0.531	0.407	0.826	0.421	0.697	0.58	0.123	0.489	0.973	0.277	0.903	0.643	0.793
EMA	*M*_IN-OUT_	0.07	−0.04	0.05	0.26	0.04	0.03	−0.22	−0.08	−0.20	−0.04	−0.20	−0.16	−0.16	−0.17
	*p*	0.666	0.764	0.671	0.061	0.792	0.878	0.252	0.685	0.274	0.841	0.365	0.408	0.547	0.446

Note. T1–T14: 100 ms time-bins from 100 to 1400 ms post-stimulus. EMH = emotional mimicry to happy faces. EMA = emotional mimicry to angry faces. *M_IN-__OUT_* = the mean difference between in-group and out-group conditions.

## Data Availability

The data presented in this study are available on request from the corresponding author. The data are not publicly available due to privacy and ethical restrictions.

## References

[1] Hess U., Fischer A. (2013). Emotional Mimicry as Social Regulation. Pers. Soc. Psychol. Rev..

[2] Stel M., Hess U., Fischer A. (2016). The role of mimicry in understanding the emotions of others. Emotional Mimicry in Social Context.

[3] Cattaneo L., Veroni V., Boria S., Tassinari G., Turella L. (2018). Sex differences in affective facial reactions are present in childhood. Front. Integr. Neurosci..

[4] de Klerk C.C., Hamilton A.F.D.C., Southgate V. (2018). Eye contact modulates facial mimicry in 4-month-old infants: An EMG and fNIRS study. Cortex.

[5] Hühnel I., Kuszynski J., Asendorpf J.B., Hess U. (2018). Emotional mimicry of older adults’ expressions: Effects of partial inclusion in a Cyberball paradigm. Cogn. Emot..

[6] Isomura T., Nakano T. (2016). Automatic facial mimicry in response to dynamic emotional stimuli in five-month-old infants. Proc. Royal Soc. B.

[7] Slessor G., Bailey P.E., Rendell P.G., Ruffman T., Henry J.D., Miles L.K. (2014). Examining the time course of young and older adults’ mimicry of enjoyment and nonenjoyment smiles. Emotion.

[8] Larsen J.T., Norris C.J., Cacioppo J.T. (2003). Effects of positive and negative affect on electromyography activity over zygomaticus major and corrugator supercilii. Psychophysiology.

[9] Hess U., Fischer A. (2014). Emotional mimicry: Why and when we mimic emotions. Soc. Personal. Psychol. Compass.

[10] Hess U., Fischer A.H. (2016). . Emotional Mimicry in Social Context.

[11] Seibt B., Mühlberger A., Likowski K., Weyers P. (2015). Facial mimicry in its social setting. Front. Psychol..

[12] Kraaijenvanger E.J., Hofman D., Bos P.A. (2017). A neuroendocrine account of facial mimicry and its dynamic modulation. Neurosci. Biobehav. Rev..

[13] Bourgeois P., Hess U. (2008). The impact of social context on mimicry. Biol. Psychol..

[14] Van Der Schalk J., Fischer A., Doosje B., Wigboldus D., Hawk S., Rotteveel M., Hess U. (2011). Convergent and divergent responses to emotional displays of ingroup and outgroup. Emotion.

[15] Forsyth D.R. (2019). Group Dynamics.

[16] Tajfel H., Turner J., Austin W., Worchel S. (1979). An integrative theory of intergroup conflict. the Social Psychology of Intergroup Relations.

[17] Turner J.C., Hogg M.A., Oakes P.J., Reicher S.D., Wetherell M.S. (1987). Rediscovering the Social Group: A Self-Categorization Theory.

[18] Turner J.C., Brown R.J., Tajfel H. (1979). Social comparison and group interest in ingroup favouritism. Eur. J. Soc. Psychol..

[19] Leach C.W., Van Zomeren M., Zebel S., Vliek M.L., Pennekamp S.F., Doosje B., Ouwerkerk J.W., Spears R. (2008). Group-level self-definition and self-investment: A hierarchical (multicomponent) model of in-group identification. J. Pers. Soc. Psychol..

[20] Enock F.E., Tipper S.P., Over H. (2021). Intergroup preference, not dehumanization, explains social biases in emotion attribution. Cognition.

[21] Krautheim J.T., Dannlowski U., Steines M., Neziroğlu G., Acosta H., Sommer J., Benjamin S., Kircher T. (2019). Intergroup empathy: Enhanced neural resonance for ingroup facial emotion in a shared neural production-perception network. Neuroimage.

[22] Over H. (2018). The influence of group membership on young children’s prosocial behaviour. Curr. Opin. Psychol..

[23] Cikara M., Van Bavel J.J., Ingbretsen Z.A., Lau T. (2017). Decoding “us” and “them”: Neural representations of generalized group concepts. J. Exp. Psychol. Gen..

[24] Gamond L., Vilarem E., Safra L., Conty L., Grèzes J. (2017). Minimal group membership biases early neural processing of emotional expressions. Eur. J. Neurosci..

[25] Wang Y., Zhang Z., Bai L., Lin C., Osinsky R., Hewig J. (2017). Ingroup/outgroup membership modulates fairness consideration: Neural signatures from ERPs and EEG oscillations. Sci. Rep..

[26] Brown L.M., Bradley M.M., Lang P.J. (2006). Affective reactions to pictures of ingroup and outgroup members. Biol. Psychol..

[27] Sachisthal M.S., Sauter D.A., Fischer A.H. (2016). Mimicry of ingroup and outgroup emotional expressions. Compr. Results. Soc. Psychol..

[28] Rauchbauer B., Majdandžić J., Stieger S., Lamm C. (2016). The modulation of mimicry by ethnic group-membership and emotional expressions. PLoS ONE.

[29] Achaibou A., Pourtois G., Schwartz S., Vuilleumier P. (2008). Simultaneous recording of EEG and facial muscle reactions during spontaneous emotional mimicry. Neuropsychologia.

[30] Kuang B.B., Li X.T., Li X.T., Lin M.X., Liu S.R., Hu P. (2021). The effect of eye gaze direction on emotional mimicry: A multimodal study with electromyography and electroencephalography. NeuroImage.

[31] Rauchbauer B., Majdandžić J., Hummer A., Windischberger C., Lamm C. (2015). Distinct neural processes are engaged in the modulation of mimicry by social group-membership and emotional expressions. Cortex.

[32] Luck S.J. (2014). An Introduction to the Event-Related Potential Technique.

[33] Bai L., Ma H., Huang Y.X., Luo Y.J. (2005). The Development of Native Chinese Affective Picture System-A pretest in 46 College Students. Chin. Ment. Health J..

[34] Tottenham N., Tanaka J.W., Leon A.C., McCarry T., Nurse M., Hare T.A., Marcus D.J., Westerlund A., Casey B.J., Nelson C. (2009). The NimStim set of facial expressions: Judgments from untrained research participants. Psychiatry Res..

[35] Krumhuber E.G., Tamarit L., Roesch E.B., Scherer K.R. (2012). FACSGen 2.0 animation software: Generating three-dimensional FACS-valid facial expressions for emotion research. Emotion.

[36] Bradley M.M., Lang P.J. (1994). Measuring emotion: The self-assessment manikin and the semantic differential. J. Behav. Ther. Exp. Psychiatry..

[37] Dimberg U., Petterson M. (2000). Facial reactions to happy and angry facial expressions: Evidence for right hemisphere dominance. Psychophysiology.

[38] Fridlund A.J., Cacioppo J.T. (1986). Guidelines for human electromyographic research. Psychophysiology.

[39] Murata A., Saito H., Schug J., Ogawa K., Kameda T. (2016). Spontaneous facial mimicry is enhanced by the goal of inferring emotional states: Evidence for moderation of “automatic” mimicry by higher cognitive processes. PLoS ONE.

[40] Sonnby-Borgström M., Jönsson P., Svensson O. (2003). Emotional empathy as related to mimicry reactions at different levels of information processing. J. Nonverbal. Behav..

[41] Oostenveld R., Praamstra P. (2001). The five percent electrode system for high-resolution EEG and ERP measurements. Clin. Neurophysiol..

[42] Kim B., Kim L., Kim Y.H., Yoo S.K. (2017). Cross-association analysis of EEG and EMG signals according to movement intention state. Cogn. Syst. Res..

[43] Likowski K.U., Mühlberger A., Gerdes A.B.M., Wieser M.J., Pauli P., Weyers P. (2012). Facial mimicry and the mirror neuron system: Simultaneous acquisition of facial electromyography and functional magnetic resonance imaging. Front. Neurosci..

[44] Charness G., Gneezy U., Kuhn M.A. (2012). Experimental methods: Between-subject and within-subject design. J. Econ. Behav. Organ..

[45] Kawakami K., Friesen J., Vingilis-Jaremko L. (2018). Visual attention to members of own and other groups: Preferences, determinants, and consequences. Soc. Personal..

[46] Xie Y., Zhong C., Zhang F., Wu Q. The ingroup disadvantage in the recognition of micro-expressions. Proceedings of the 2019 14th IEEE International Conference on Automatic Face & Gesture Recognition.

[47] Tajfel H. (1982). Social psychology of intergroup relations. Annu. Rev. Psychol..

[48] Chartrand T.L., Lakin J.L. (2013). The antecedents and consequences of human behavioral mimicry. Annu. Rev. Psychol..

[49] Fischer A., Hess U. (2017). Mimicking emotions. Curr. Opin. Psychol..

[50] Wang Y., Hamilton A.F.D.C. (2012). Social top-down response modulation (STORM): A model of the control of mimicry in social interaction. Front. Hum. Neurosci..

[51] Colombatto C., McCarthy G. (2017). The effects of face inversion and face race on the P100 ERP. J. Cogn. Neurosci..

[52] Cunningham W., Van Bavel J., Arbuckle N., Packer D., Waggoner A. (2012). Rapid social perception is flexible: Approach and avoidance motivational states shape P100 responses to other-race faces. Front. Hum. Neurosci..

[53] Ito T.A., Bartholow B.D. (2009). The neural correlates of race. Trends Cogn. Sci..

[54] Herrmann M.J., Ehlis A.C., Ellgring H., Fallgatter A.J. (2005). Early stages (P100) of face perception in humans as measured with event-related potentials (ERPs). J. Neural Transm..

[55] Vogel E.K., Luck S.J. (2000). The visual N1 component as an index of a discrimination process. Psychophysiology.

[56] Wang H., Guo S., Fu S. (2016). Double dissociation of configural and featural face processing on P1 and P2 components as a function of spatial attention. Psychophysiology.

[57] Balconi M., Lucchiari C. (2007). Consciousness and emotional facial expression recognition: Subliminal/supraliminal stimulation effect on N200 and P300 ERPs. J. Psychophysiol..

[58] Luck S.J., Kappenman E.S. (2011). The Oxford Handbook of Event-Related Potential Components.

[59] Fredickson B.L. (2003). The value of positive emotions. Am. Sci..

